# A multimeal paradigm producing a low glycemic response is associated with modest cognitive benefits relative to a high glycemic response: a randomized, crossover trial in patients with type 2 diabetes

**DOI:** 10.1016/j.ajcnut.2023.02.017

**Published:** 2023-02-24

**Authors:** Matthew Grout, Julie A. Lovegrove, Daniel J. Lamport

**Affiliations:** 1 School of Psychology and Clinical Language Science, University of Reading, United Kingdom; 2 Hugh Sinclair Unit of Human Nutrition and Institute for Cardiovascular and Metabolic Research, Institute of Food, Nutrition and Health, Department of Food and Nutrition Sciences, University of Reading, United Kingdom

**Keywords:** glycemic index, glycemic load, cognition, type 2 diabetes, glucose

## Abstract

**Background:**

Type 2 diabetes (T2DM) and poor glucose regulation in the immediate postprandial period are both associated with impairments in cognitive function. There is evidence that foods that generate a better postprandial glycemic response, such as low GI foods (which produce a lower glycemic peak, less variability, and a more sustained decline), are associated with cognitive benefits over the morning. However, the potential impact of consuming multiple meals of this nature over the course of a day on cognition in T2DM has not been explored.

**Objectives:**

The primary aim of this research was to investigate whether a multimeal paradigm producing a low glycemic response was associated with cognitive benefits in patients with noninsulin-dependent T2DM relative to a multimeal paradigm producing a high glycemic response.

**Methods:**

Twenty-five adults with noninsulin-dependent T2DM (mean age: 57 y) consumed 2 multimeal profiles consisting of a breakfast, lunch, and afternoon snack on 2 separate test days following a randomized, counterbalanced, crossover design. The 2 conditions were a low GI profile (LGIP) and a high GI profile (HGIP).

**Results:**

Cognitive function, glycemic response, mood, and satiety were assessed over the day from 8:30 to 17:00. Overall, there were limited cognitive effects. However, there was evidence for cognitive benefits in the period before lunch, as demonstrated by better global cognitive and executive functions for the LGIP relative to the HGIP. No clear effects were observed for mood.

**Conclusions:**

This study shows that a multimeal paradigm producing a low glycemic response was associated with some benefits for cognitive function in patients with T2DM.

**Clinical Trail Registry reference:**

NCT03360604 (clinical trial.gov).

## Introduction

Given that glucose is the main fuel for the brain, it is perhaps not surprising that conditions that are associated with abnormalities in glucose regulation, such as type 2 diabetes (T2DM), are associated with cognitive impairments [1]. Greater severity of T2DM, defined by higher HbA1C concentrations and more frequent hyper- and hypoglycemic episodes, are associated with greater decrements in cognitive function and increased risk of neurodegenerative diseases such as dementia and Alzheimer’s disease [2]. Furthermore, interventions such as increased physical activity, which can maintain or improve glycemic control, have been associated with beneficial effects on cognitive function in patients with T2DM, although reviews indicate that the evidence is mixed and effect sizes are small [3,4]. In patients with noninsulin-dependent T2DM, the most significant daily contributor to the regulation of glucose concentrations is dietary intake. A number of studies have explored whether foods that generate a glycemic response with less variability over the post-prandial period, including lower peaks, fewer troughs, and a steadier prolonged decline, can benefit cognitive function [5]. For example, improved cognitive performance in patients with T2DM and adults with impaired glucose tolerance (prediabetes) has been observed over the course of the morning following consumption of low GI foods relative to high GI foods [6,7].

Interestingly, dietary intervention studies that have investigated the link between glycemic response and cognitive performance in T2DM have used a single meal paradigm, typically focusing on breakfast. However, humans consume multiple meals throughout the day, spending most of any day in a postprandial state. Therefore, it is more representative of everyday dietary habits if a multiple meal protocol is used to determine the effects of dietary manipulations to improve glycemic control on postprandial cognitive function. The second meal effect demonstrates that the glycemic response to a previous meal can influence the glycemic response to the next meal. This mechanism has also been demonstrated for cognition whereby the nature of the evening meal can impact cognition the next day following an overnight fast, even after a standardized breakfast; a process known as the second meal cognitive effect [8]. Therefore, it follows that multiple meals, which generate a glycemic response with less variability over the course of a single day, maybe hypothesized to benefit cognitive function relative to multiple meals associated with greater glycemic variability (an unfavorable glycemic response). However, this concept has not yet been investigated.

Cognitive function is also impacted by mood state, and in some cases, the mechanism for cognitive benefits can be via changes to mood states such as improved self-reported alertness and higher levels of contentment. Although the primary outcome measure here is cognitive function, mood state is also assessed as a secondary outcome measure. Indeed, there is some evidence that a low GI (LGI) diet and LGI foods are beneficial for mood outcomes relative to high GI (HGI) [[9], [10], [11]]. Furthermore, T2DM is associated with worse mood outcomes, such as higher rates of anxiety and depression [12,13], making mood a pertinent outcome measure. Finally, foods that generate a lower glycemic response relative to energy-matched foods producing a higher glycemic response have been associated with higher levels of postprandial fullness and reduced hunger [14,15], both of which are subjective sensations that may impact mood state and cognitive function. Therefore, measures of satiety were considered as an additional secondary outcome measure. To summarize, the primary aim of this research was to investigate whether a multimeal paradigm with LGI foods consumed over the course of the day producing a low glycemic response was associated with cognitive benefits in patients with noninsulin-dependent T2DM relative to a multimeal paradigm with HGI foods. The secondary aims were to explore the effects on measures of subjective mood and satiety. It was hypothesized that the LGI glycemic profile would be associated with better cognitive, mood, and satiety outcomes relative to the HGI glycemic profile, although there is no data on which to base a hypothesis regarding the specific point(s) in the day at which these benefits may occur.

## Method

### Participants

Twenty-five adults with a medical diagnosis of T2DM were recruited through a local advertisement at the University of Reading and the surrounding areas. These included 17 men and 8 women, with a mean age of 56.9 y (SD = 7.8), a mean BMI of 30.6 kg/m^2^ (SD = 5.3), and a mean overnight fasting glucose concentration at screening of 8.44 mmol/L (SD = 2.65). Inclusion criteria were aged between 40–70 y old. All participants were self-reported nonsmokers with no relevant food intolerances or allergies. Exclusion criteria were cancer, any other condition that could affect glucose metabolism (for example, anemia and pregnancy), and currently taking antidepressants on account of effects on cognitive function. There were no dropouts. A power analysis was conducted using Gpower 3.1 to determine the sample size required. Using an effect size of *d* = 0.44 [6] with a statistical power of 0.8 and an alpha level of 0.05, 25 participants were sufficient to detect cognitive performance differences between the 2 conditions across 8 test points. Papanikolaou et al.’s [6] study was used to calculate the effect size because it is one of the few studies to examine cognitive performance exclusively in patients with T2DM after the consumption of HGI and LGI meals. The effect size was based on a verbal memory measure (the only outcome measure), which is a limitation of this power calculation, given that verbal memory is not assessed in the present study (rationale in DISCUSSION). The number of participants recruited for screening was not monitored. In total, 29 participants were randomly selected after the completion of the screening, and 14 were randomly assigned to start with the LGIP. Four participants withdrew before starting the first test day (2 from the LGI and 2 from the HGI group). In all cases, the reason for withdrawal was no longer interested in taking part. Twenty-five participants completed the study, and data analysis was conducted with all 25 participants.

### Design

**FIGURE 1:**
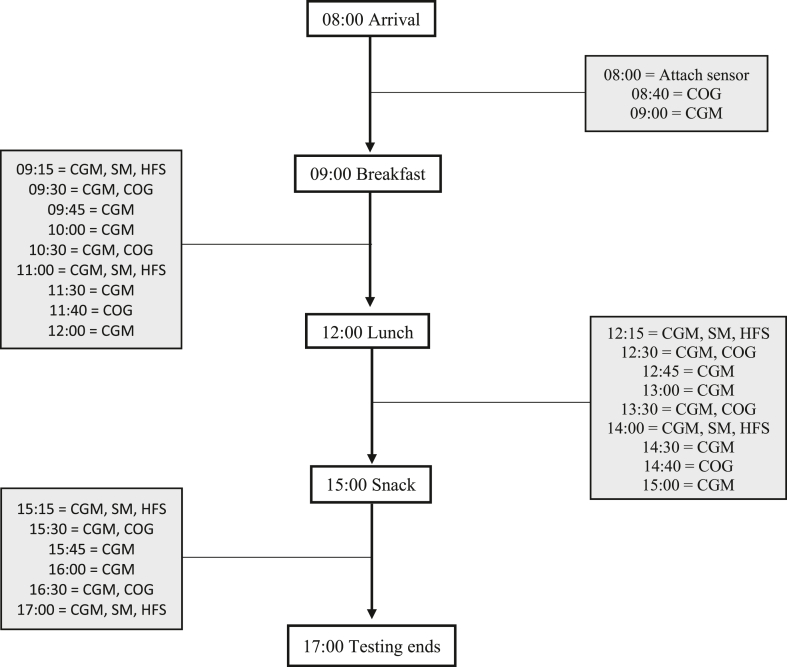
Procedural outline for both conditions. CGM, continuous glucose monitor reading; COG, cognitive performance assessment; HFS, hunger, fullness, sleepiness evaluation; SM, subjective mood evaluation.

The study followed a counterbalanced, randomized, crossover design with 2 nutritional interventions that produced 2 different glycemic profiles (2 conditions); (1) a low GI profile (LGIP) and (2) a high GI glycemic profile (HGIP). Both conditions consisted of breakfast, lunch, and an afternoon snack (details below), and there were 9 points of assessment over the day, defined using the variable name time. The initial assessment timepoint was undertaken in a fasted state and was treated as baseline data. Eight subsequent time points took place over the course of 7.5 h (Figure 1). Condition order was randomly determined with an online randomizer (Research Randomizer https://www.randomizer.org/), which resulted in *n* = 12 beginning with the LGIP condition. Independent variables were condition and time, although broadly speaking, the primary dependent variable (DV) was cognitive performance. The 4 specific primary outcome measures were as follows: (1) choice reaction time (CRT) performance; (2) Rapid Visual Information Processing performance; (3) Letter Memory Performance; (4) performance one—the merged CRT and Rapid Visual Information Processing task. Secondary dependent variables were (i) glycemic response; (ii) mood (specifically alertness, anxiety, and contentment); (iii) sleepiness; (iv) hunger, and (v) fullness.

The 2 novel nutritional conditions (Table 1) were designed and tested in a pilot study [16]. The GI concept was utilized to produce an LGIP that was steady, with low peaks, whereas the HGIP was designed to produce higher peaks and greater variability. Both conditions were isoenergetic (1310kcal), with each meal being matched for energy and macronutrients. According to the Foster-Powell et al. [17,18] method, the glycemic load of the LGIP and HGIP was 61.5 and 79.4, respectively. One of the authors (MG) generated the random allocation sequence, enrolled participants, and assigned participants to interventions.

**TABLE 1:** Macronutrient composition, GI, and glycemic load (GL) calculations for the low GI profile (LGIP) and high GI profile (HGIP) (*n* = 25)

Low GI profile	Weight (g)	Fat (g)	Protein (g)	CHO (g)	Energy (kcal)	Fiber (g)	GI	PCF (%)	GI ∗ PCF/100	GI ∗ CHO/100
High bran cereal	29	1	4.1	13.9	96.9	7.8	44	26.5	11.7	6.1
Skimmed milk	126	0.1	4.3	6.3	44.1	0	48	12	5.8	3
Apple juice	226	0.2	0	26.4	106.2	0.2	40	50.3	20.1	10.6
Yogurt	84	1.2	4.2	5.9	51.2	0	35	11.2	3.9	2.1
**Breakfast total**	**465**	**2.5**	**12.5**	**52.5**	**298.4**	**8**		**GI**	**41.5**	**GL 21.7**
Pasta bake	440	25.9	23.7	87.5	699.6	10.1	23	100	23	20.1
**Lunch total**	**440**	**25.9**	**23.7**	**87.5**	**699.6**	**10.1**		**GI**	**23**	**GL 20.1**
Raw apple	133	0.1	0.5	15.7	70.5	2.4	32	29.2	9.3	5
Cashew nuts	17	8.2	3.3	4.1	104.7	0.6	27	7.6	2.1	1.1
Apple juice	290	0.3	0	33.9	136.3	0.3	40	63.2	25.3	13.6
**Snack total**	**440**	**8.6**	**3.8**	**53.7**	**311.5**	**3.3**		**GI**	**36.6**	**GL 19.7**

**High GI profile**	Weight (g)	Fat (g)	Protein (g)	CHO (g)	Energy (kcal)	Fiber (g)	GI	PCF (%)	GI ∗ PCF/100	GI ∗ CHO/100
Flakes of corn	30	0.3	2.1	25.2	113.4	0.9	93	47.3	44	23.4
Skimmed milk	220	0.2	7.5	11	77	0.9	48	20.6	9.9	5.3
White bread	38	0.7	3.3	17	88.5	0	75	31.8	23.9	12.7
Margarine spread	3	2.1	0	0	18.9	0	0	0.3	0	0
**Breakfast total**	**291**	**3.3**	**12.9**	**53.2**	**297.8**	**1.8**		**GI**	**77.8**	**GL 41.4**
White bread	76	1.3	6.6	33.9	177.1	1.8	75	38.3	28.7	25.4
Soft cheese spread	79	8.7	5.9	4	120.1	0.4	0	4.5	0	0
Cheddar cheese	46	16.1	11.7	0.1	191.4	0	0	0.2	0	0
Lettuce	40	0.2	0.3	0.7	6.4	0.4	15	0.7	0.1	0.1
Lucozade, original	293	0	0	49.8	205.1	0	95	56.3	53.5	47.3
**Lunch total**	**534**	**26.3**	**24.5**	**88.5**	**700.1**	**2.6**		**GI**	**82.3**	**GL 72.8**
Jelly candy	28	0.1	0.1	27.3	106.4	0	80	51.1	40.9	21.9
Lemon curd yogurt	105	9.4	3.7	17.8	170.1	0.2	67	33.1	22.2	11.9
Glucose drink	50	0	0	8.5	35	0	95	15.8	15	8.1
**Snack total**	**183**	**9.5**	**3.8**	**53.6**	**311.5**	**0.2**		**GI**	**78.1**	**GL 41.9**

TMC, total meal carbohydrate; PCF, proportion of carbohydrate from each food. PCF = CHO/TMC × 100. GI values are taken from Atkinson et al. [17].

### Procedure

All exclusion criteria were checked with a self-report questionnaire which participants completed and returned by e-mail prior to a screening session. A 1-h screening session was arranged for the morning at the Hugh Sinclair Unit of Human Nutrition, University of Reading, when height and weight were measured with a Tanita BC-418MA body composition monitor (TANITA Corporation). A venous blood sample was collected in a fasting state, and serum glucose was determined using an Accu-Chek Aviva Blood Glucose Meter System (Roche Diagnostics). A practice run of the cognitive task battery was completed (data not collected) as recommended by Bell et al. [19]. The first test day was arranged ≥1 wk after the screening session, with further 7 d between test day 1 and test day 2. For 24 h prior to each test visit, participants were asked to refrain from the consumption of alcohol and avoid any form of exercise. For the evening prior to each test day, a standardized meal was provided for consumption at home between 18:00 and 20:00 consisting of 2 slices of white bread and a tin of beans in tomato sauce. After the evening meal, participants were required to fast (no food or drink except water) for the rest of the day/following morning.

For each test day, upon arrival at 08:00, participants had a continuous glucose sensor (FreeStyle Libre Abbott Diabetes Care Inc) attached to the back of their upper left arm. While the sensor self-calibrated, the participant waited in a quiet room where they were able to watch television or read materials provided within the High Sinclair Research Unit. As shown in Figure 1, the test meals were administered at 09:00, 12:00, and 15:00 to mimic representative meal times found in a habitual diet. The first cognitive (baseline) assessment began at 08:40, followed by an interstitial glucose reading being taken immediately before the consumption of breakfast at 09:00. Participants were required to consume all of the test meals within 15 min. The cognitive battery implemented in this study lasted ∼20 min. Cognitive assessments were initiated 20 min prior to each meal, then 30, 90, and 160 min post meal serving (there was no assessment 160 min post snack meal). A total of 23 glucose readings were taken throughout the day, with a reading taken immediately before each meal (0 min) and 15, 30, 45, 60, 90, 120, and 150 min post meal consumption. Subjective mood and satiety were self-evaluated at 15 and 120 min post meal consumption. The test day ended at 17:00. Participants were remunerated for their time and travel expenses upon completion of the study. For all test days, participants were instructed to continue with their normal regime for prescribed medications to comply with the ethical requirements that the study would have no impact on medication regimes. Data relating to the intake of medications was not collected. This study was approved by the School of Psychology Research Ethics Committee (SREC 2017-151-DL), the University of Reading Research Ethics Committee (UREC 17/63), and the East of Scotland Research Ethics Service (EoSRES IRAS 237190). The study was registered on clinical trial.gov (NCT03360604).

#### Cognitive function

The cognitive task battery was administered with E-Prime 2.0 (Psychology Software Tool, Inc). There were 4 separate cognitive tasks: CRT, Rapid Visual Information Processing (RVIP), a merged CRT-RVIP task, and Letter Memory. The CRT task was a measure of general alertness and psychomotor speed. For each trial, a fixation “x” appeared in the center of the screen, which was replaced by a target X either to the left or right of the fixation x. Participants were required to indicate whether the target appeared to the left or right of the fixation point by pressing the relevant key (z or m, respectively) as quickly as possible. The interstimulus interval that separated each trial ranged from 250 ms to 1500 ms in a random fashion (matched across versions). This task lasted for ∼3 min with a total of 60 targets presented. The DVs were accuracy score (maximum 60) and mean reaction time (ms) for correct responses.

The RVIP was a measure of sustained attention and working memory. During this task, participants were presented with a continuous string of single numerical digits ranging from 1 to 9 in the center of the screen. The string of numbers was presented at a rate of 75 digits/min, with each trial fixed at 800 ms. Participants continuously monitored the digits for 2 specific target strings, which were “1, 3, and 5” and “6, 4, and 2”. When a target string was observed, the participant would press the space bar as quickly as possible. This task lasted ∼4 min, with a total of 270 single digits being presented, including 20 target strings. The DVs for the task were accuracy score (maximum 20) and mean reaction time (ms) for correct responses.

The merged task was a novel concept designed to increase cognitive effort by combining the testing parameters of the CRT and RVIP tasks. Thus, this task is a measure of sustained attention, working memory, and psychomotor speed. The rationale for this task is that it combines 2 already standardized tasks; therefore, the level of demand can be evaluated by comparing the performance of the merged task with the performance of the individual tasks. By combining 2 simpler tests into a more difficult task, the effect of increasing task difficulty can be explored, as previous studies have shown that more difficult demanding tasks are more sensitive to the effects of glucose regulation [20,21]. Throughout this task, each trial contained 2 aspects: (1) a single digit in the center of the screen and (2) a target “x” that appeared to the left or right of the central digit. Both aspects continuously changed between trials in a pseudorandom fashion. The participant was required to press the relevant key (z or m) to indicate which side the “x” had appeared on every trial and simultaneously press the space bar if either target string (1, 3, and 5 or 6, 4, and 2) was observed. Each trial had a fixed duration of 800 ms, and the interstimulus interval that separated each trial ranged randomly from 200 ms to 1000 ms. This task lasted ∼8 min, with a total of 270 trials being presented, including 250 CRT-only trials and 20 combined task trials. The dependent variables for the task were accuracy scores on combined task trials (maximum 20) as well as mean reaction time (ms) for correct responses. The rationale for 20 target trials on the merged CRT-RVIP task was to match the number of targets on the RVIP-only task to allow a direct comparison of outcomes between the RVIP and merged CRT-RVIP task.

The Letter Memory task was a measure of executive function. During this task, participants were presented with a series of letters (consonants only) that appeared individually in the center of the screen. The number of letters presented was either 5 or 7 (8 of each), which randomly varied. When a sequence of letters had ended, participants were presented with a screen that displayed 4 options. Participants had to press the relevant button (1, 2, 3, or 4) to indicate which option contained the last 4 letters that had appeared. Once the participant had indicated their choice, the next sequence of letters would begin. The series of letters were presented at a rate of 30 letters/min, with each letter appearing for 2000 ms. At the end of each sequence, the participant had a maximum of 8000 ms to indicate their choice of the 4 options presented. If they made no choice during the 8000 ms, the next sequence would begin, and no selection was recorded. This task lasted for ∼5 min, with a total of 96 letters being presented across 16 separate sequences. The dependent variables for the task were accuracy score (maximum 16) and mean reaction time (ms) for correct responses.

Alternate forms of all cognitive tests were counterbalanced across the test days and time points.

#### Glycemic response

Glucose concentrations were measured using a FreeStyle Libre continuous glucose monitoring system (Abbott Diabetes Care Inc). The sensor automatically measured interstitial glucose concentrations every minute and stored readings at 15-min intervals for 8 h. The data was wirelessly transmitted to the reader held by the experimenter upon scanning. During each test day, a total of 23 interstitial glucose readings were taken; immediately before each meal (0 min) and 15, 30, 45, 60, 90, 120, and 150 min post meal consumption (Figure 1). This procedure allowed glucose measurements to be taken regularly without interrupting meal consumption or cognitive performance and subjective mood assessments at the end of a test day; the sensor was removed from the participant’s arm by the researcher. Previous research has reported interstitial glucose measurements with the FreeStyle Libre system are accurate compared with capillary blood glucose reference values and remain accurate over 14 d of wear in people with type 1 and 2 diabetes [22]. The initial baseline fasting glucose concentration at screening each test day was taken with a capillary blood sample via finger pricks using the Accu-Chek Aviva Blood Glucose Meter System (Roche Diagnostics).

#### Subjective mood and satiety

The Bond-Lader mood questionnaire [23] was administered in paper form as per the schedule shown in Figure 1. The questionnaire presents participants with 16 individual lines, with each line having opposing mood-related adjectives at either end. To indicate their current mood in relation to the adjectives, the participant would mark each line with a pen nearest the adjective that represented their feelings at the present moment. Each line had a length of 100 mm, resulting in a recordable score of 0 to 100 for each pairing of mood-related adjectives. Weighted scores for adjective pairings were then combined according to published criteria [23], resulting in 3 mood subfactors: alertness, anxiety, and contentment. Higher ratings in these subfactors indicated higher levels of alertness, anxiety, or contentment. Subjective measurements of hunger, fullness, and sleepiness were captured with visual analog scales in an identical manner to the Bond-Lader mood scales, with the adjectives “not at all” and “very” used as the anchor points at each end of the scale. Higher self-reported ratings indicated higher subjective levels of hunger, fullness, or sleepiness.

#### Analysis

Linear mixed models were used for all analyses. For glycemic response, the independent variables condition (LGIP and HGIP) and time (22-time points) were included as fixed factors, with the following covariates included as fixed factors; sex, age, BMI, baseline glucose, and baseline DV score. Baseline glucose was the fasted baseline reading on the test day, and baseline DV score was a performance for the dependent variable being analyzed at the first session of the day. For the glycemic response analysis, baseline glucose and baseline DV represented the same data point, so this was only included once in the model. Additionally, time was specified as a repeated variable to control the covariance structure for each participant. The interaction condition∗time was specified in the model. Pairwise comparisons with Bonferroni corrections were embedded within the model. Participant identification (ID) was included as a random factor to control for the nonindependence of data within participants. The main effects of time are not reported here as these do not inform the research questions. Data from participants (*n* = 4) who were selected randomly but withdrew from the study prior to the first test day were excluded from the analysis. The remainder of the randomly selected participants (*n* = 25) completed the protocol and were included in the analyses. To capture overall cognitive performance across all tasks, global cognitive performance (GCP) was calculated by initially converting scores to z-scores for each test and subsequently calculating an average z-score per participant per time point per condition as documented elsewhere [24]. A higher z-score represents better performance. All analyses were conducted using IBM SPSS Statistics v24, and a p-value of 0.05 was set as statistically significant. The assumptions of normality and linearity of residuals were met based on Q-Q plot, and the assumption of homoscedasticity between the low GI and high GI conditions was met based on Levene’s tests.

## Results

Background characteristics according to the randomization sequence for this crossover trial are shown in Table 2.

**TABLE 2:** Baseline characteristics of participants by randomization sequence (either LGIP first and HGIP second or HGIP first and LGIP second) data are means and SE

	Male/female	Age (y)	Height (cm)	Weight (kg)	BMI (kg/m^2^)	HbA1c	Medication^1^/ Diet treatment
LGIP first (*n* = 12)	8/4	57 (2)	175 (3)	87 (6)	28.3 (1)	58 (6)	10/2
HGIP first (*n* = 13)	9/5	56 (2)	173 (2)	98 (5)	32.6 (1)	61 (6)	11/2

LGIP, low GI profile; HGIP, high GI profile. ^1^Includes metformin (*n* = 19), gliclazide (*n* = 6), dapagliflozin (*n* = 5), alogliptin (*n* = 2), and empagliflozin (*n* = 2).

### Glycemic response

**FIGURE 2:**
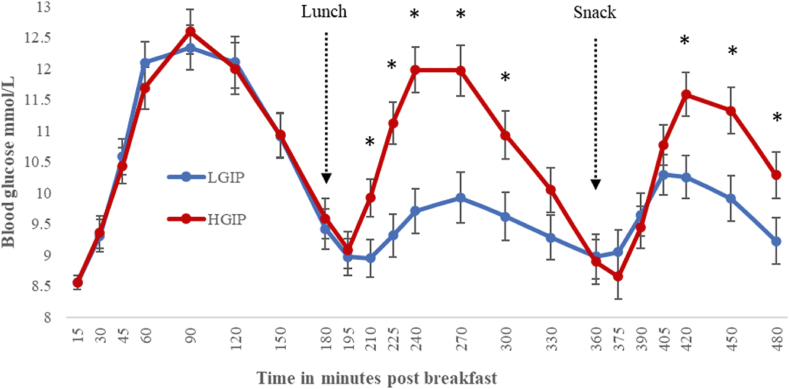
Blood glucose concentrations (mmol/L) for the low GI profile (LGIP) and the high GI meal profile (HGIP) for the test day following breakfast (*n* = 25, crossover design) collected with a continuous glucose monitor. Asterix indicates a significant difference (*P* < 0.05) between the LGIP and the HGIP based on pairwise comparisons following a significant condition × time interaction for the Linear Mixed Model [F_(21,380)_ = 4.42, *P* < 0.001] with baseline glucose, gender, age, and BMI as a covariates. Data are means and SEs.

As shown in Figure 2, the HGIP was associated with higher glucose concentrations throughout the afternoon after lunch and snack, as indicated by a significant condition∗time interaction [F_(21,380)_ = 4.42, *P* < 0.001]. Specifically, pairwise comparisons revealed that glucose was significantly higher in the HGIP condition at 210, 225, 240, 270, 300, 420, 450, and 480 min (all *P* < 0.05). There was no difference between conditions in the glycemic response to breakfast at any point over the morning. The overall main effect of the condition approached significance values (*P* = .089).

### Cognitive performance

**FIGURE 3:**
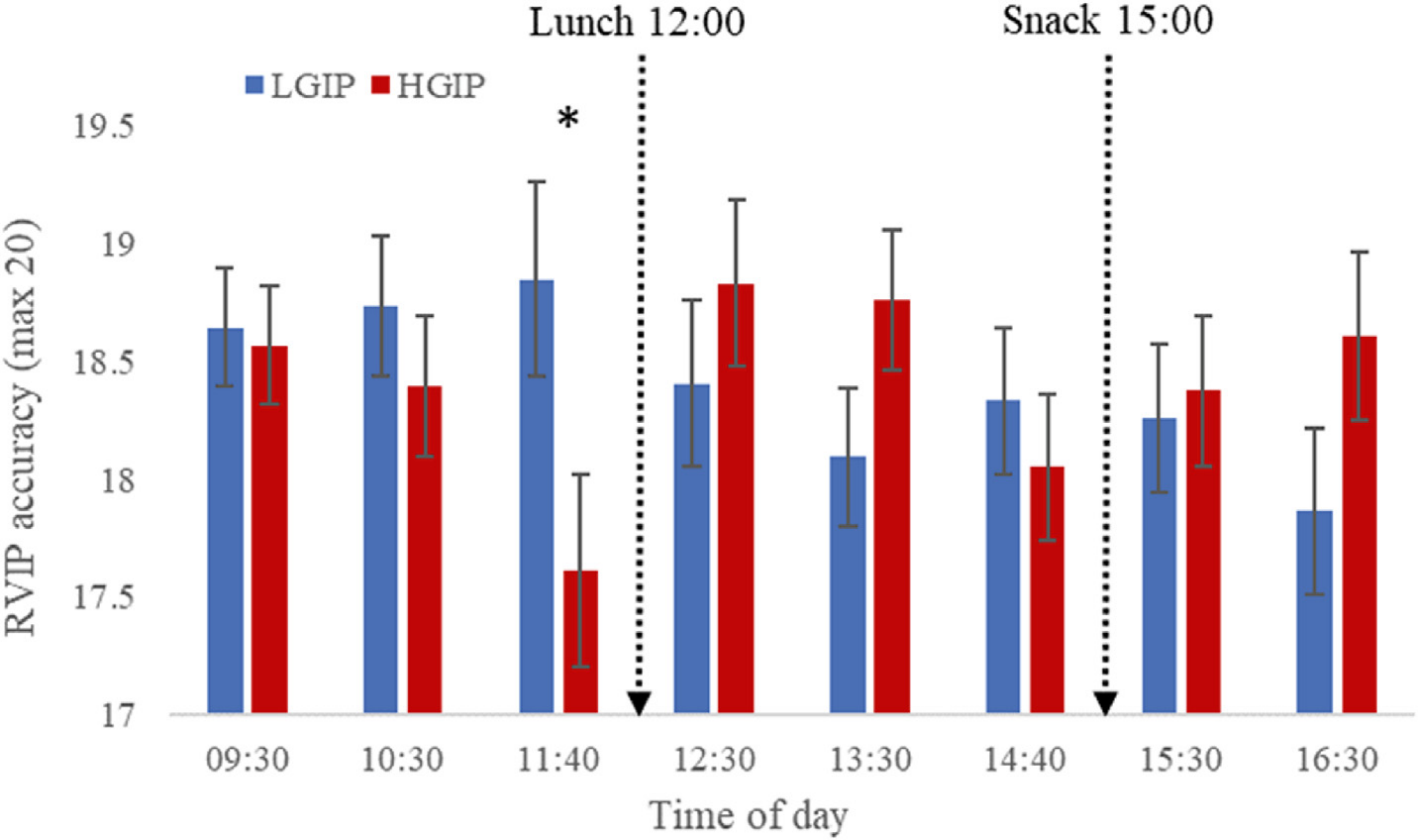
Accuracy on the RVIP task at each test session for the low GI profile (LGIP) and the high GI profile (HGIP) (*n* = 25, crossover design). Asterix indicates a significant difference *(P* < 0.05) between the LGIP and the HGIP based on pairwise comparisons following a significant condition × time interaction for the Linear Mixed Model [F_(7,50)_ = 2.36, *P* < 0.05] with baseline RVIP, gender, age, and BMI as a covariates. Data are means and SEs. Breakfast was consumed at 09:00 and the baseline session was at 08:40. RVIP, rapid visual information processing.

**FIGURE 4:**
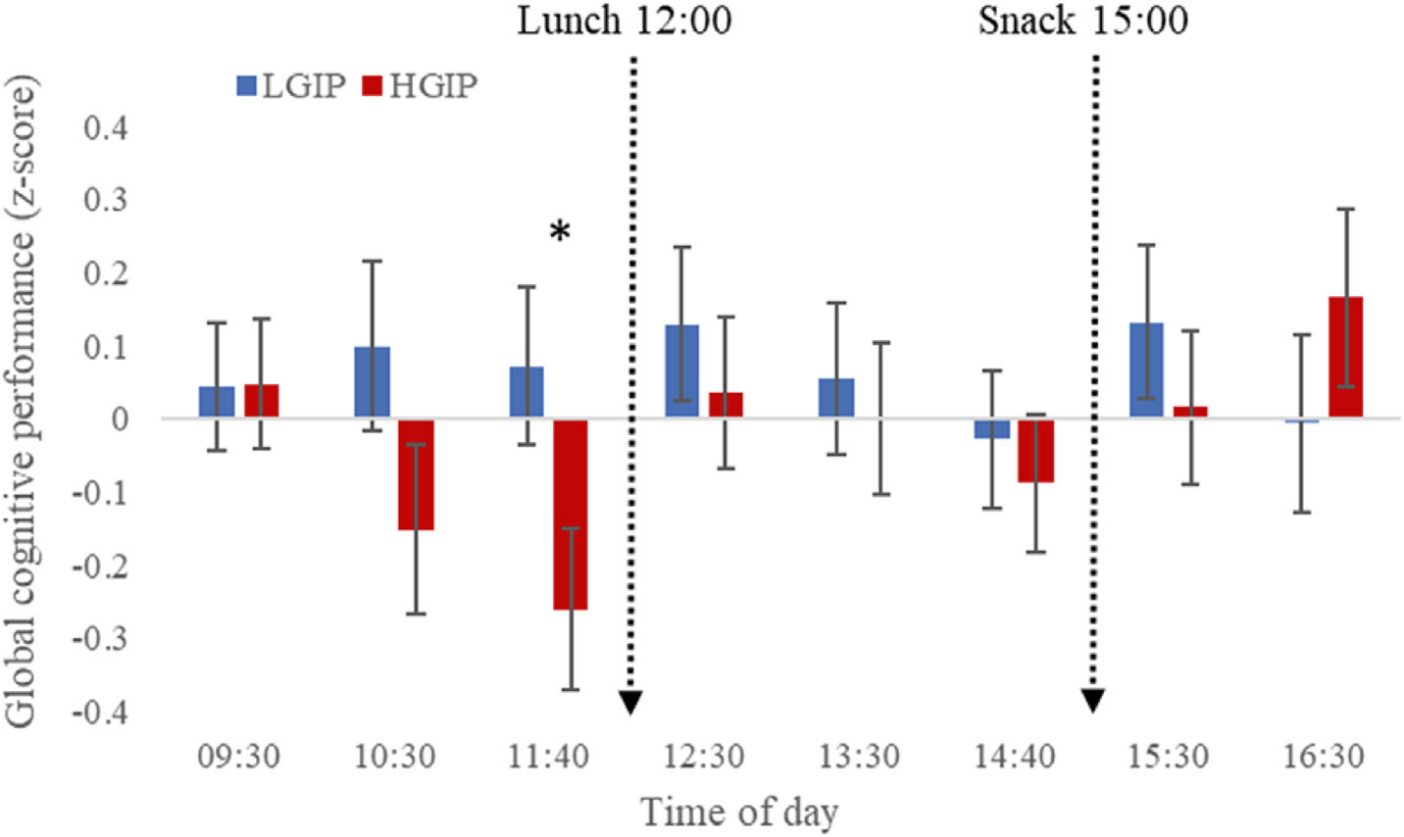
Global cognitive performance at each test session for the low GI profile (LGIP) and the high GI meal profile (HGIP) (*n* = 25, crossover design). Asterix indicates a significant difference (*P* < 0.05) between the LGIP and the HGIP based on pairwise comparisons following a significant condition × time interaction for the Linear Mixed Model [F_(7,50)_ = 2.5, *P* < 0.05] with baseline global performance, gender, age, and BMI as a covariates. Data are means and SEs for z-scores, whereby zero indicates average performance across all sessions. Breakfast was consumed at 09:00 and the baseline session was at 08:40.

Table 3 shows the means (and SEs) for each condition for each cognitive and mood outcome, in addition to the p-value for the main effect of condition and condition∗time interaction. There were no main effects of condition for any cognitive outcomes; however, there were significant condition∗time interactions for RVIP accuracy [F_(7,50)_ = 2.36, *P* < 0.05] and GCP [F_(7,50)_ = 2.5, *P* < 0.05] indicating differences between the conditions on these measures at specific timepoints during the day. As shown in Figure 3, RVIP accuracy was better for the LGIP relative to the HGIP at 11:40, which was the final test session prior to lunch, as indicated by pairwise comparisons (*P* < 0.05; mean difference: 0.33; 95% CI: 0.02, 0.65). Similarly, as shown in Figure 4, the GCP was also better for the FGP relative to the UGP at 11:40, as shown by pairwise comparisons (*P* < 0.05). No other time points were significantly different between the conditions.

**TABLE 3:** Means and SEs for each of the outcome measures including cognition function, mood, and satiety for the low GI profile (LGIP) and the high GI profile (HGIP)

Variable	LGIP	HGIP	Condition *P*	Condition × time *P*
CRT accuracy (max 60)	59.1 (.2)	59 (.2)	0.895	0.065
CRT reaction time (ms)	365 (5.3)	368 (5.3)	0.56	0.185
RVIP accuracy (max 20)	18.4 (.2)	18.4 (.2)	0.997	0.037∗
RVIP reaction time (ms)	403 (5.3)	406 (5.3)	0.783	0.241
Merged accuracy (max 20)	13.6 (.6)	13.4 (.6)	0.811	0.7
Merged reaction time (ms)	570 (8.1)	570 (7.6)	0.957	0.416
Letter memory accuracy (max 16)	10.7 (.5)	10.6 (.5)	0.717	0.513
Letter memory reaction time (ms)	2950 (93)	2959 (93)	0.944	0.203
Global performance (z-score)	.063 (.08)	−.029 (.08)	0.429	0.028∗
Alertness (0–100)	67.8 (1.9)	68.5 (1.8)	0.802	0.773
Anxiety (0–100)	23.1 (1.8)	24.7 (1.8)	0.524	0.845
Contentment (0–100)	79.9 (1.3)	79.2 (1.3)	0.683	0.177
Hunger (0–100)	24.43 (3.3)	27.26 (3.4)	0.422	0.03∗
Fullness (0–100)	66.9 (2.4)	63.5 (2.4)	0.314	0.14
Sleepiness (0–100)	39.6 (2.6)	41.1 (2.6)	0.686	0.053

CRT, choice reaction time; RVIP, rapid visual information processing. Mood and satiety data were collected with Likert scale questionnaires. Significance values are presented for the main effect of condition and the condition × time interaction for the Linear Mixed Model, which included condition (LGIP and HGIP) and time (22 time points) as fixed factors and sex, age, BMI, baseline glucose and baseline DV score as covariates. All models were embedded with Bonferroni corrections (*n* = 25).

### Mood and satiety

**FIGURE 5:**
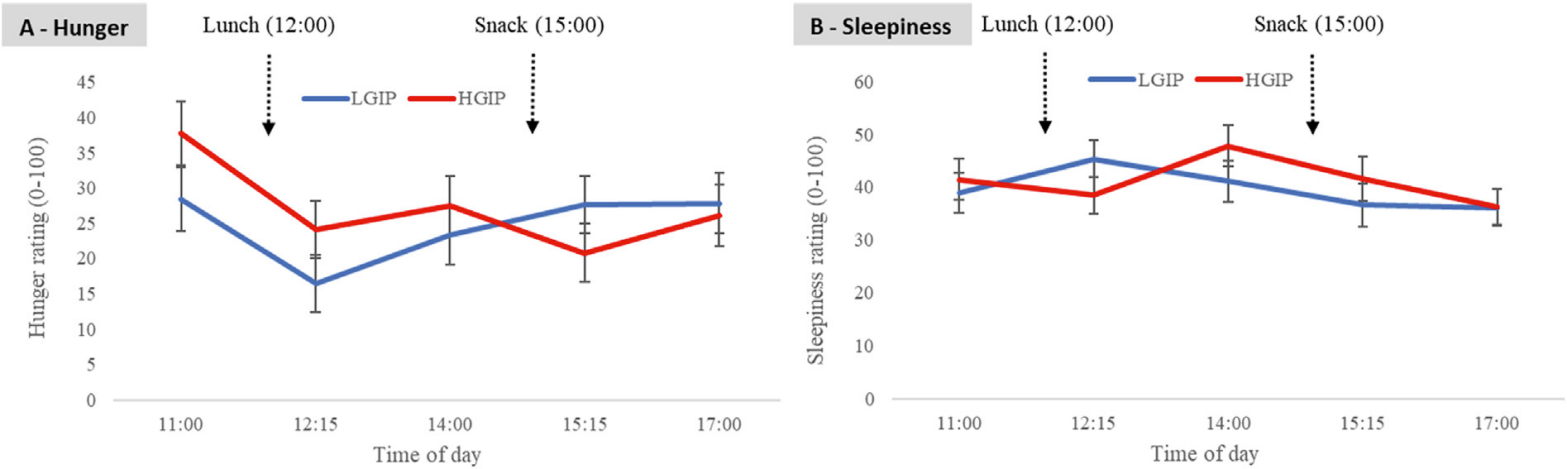
Subjective ratings of hunger (A) and sleepiness (B) for the low GI profile (LGIP) and the high GI meal profile (HGIP) (*n* = 25, crossover design). Using a Linear Mixed Model, there was a significant condition × time interactions for hunger [F_(4,50)_ = 2.36, *P* < 0.05] and a nonsignificant trend for sleepiness [F_(4,50)_ = 2.5, *P* = 0.053]; however, pairwise comparisons did not reach significance. Baseline hunger or sleepiness, gender, age, and BMI were included in the model as covariates. Data are means and SE for responses on a Likert scale with anchor points as “not at all” (score 0) and “extremely” (score 100).

As shown in Table 3, there were no main effects of condition for any mood or satiety outcomes; however, there were significant condition∗time interactions for hunger [F_(7,50)_ = 2.36, *P* < 0.05) and a nonsignificant trend for sleepiness [F_(7,50)_ = 2.5, *P* = 0.053] indicating differences between the conditions on these measures at specific timepoints during the day. Although hunger appeared to be higher for the HGIP relative to the LGIP at 11:00 and 12:15, neither of these pairwise comparisons was significant *(P >* 0.05; Figure 5A). Similarly, the pairwise comparisons for sleepiness were NS (Figure 5B).

## Discussion

The aim of this study was to explore the cognitive effects of a multimeal paradigm that produced an LGI response over the course of the day relative to meals which produced an HGI response in patients with noninsulin-dependent T2DM. Overall, there were no cognitive effects for 7 of the 9 cognitive performance measures. However, there were modest cognitive benefits in the period before lunch (2.5–3 h after breakfast), as demonstrated by better global cognitive function for the LGIP relative to the HGIP and specifically, better accuracy for the RVIP task, a measure of executive function. As a combination of all the cognitive tests, the global outcome offers insight into the general direction of effects across the board. Therefore, these results indicate that there is some acute cognitive benefit for patients with T2DM when following meal patterns that are associated with a steadier glycemic response, such as lower peaks and a tapered decline over the postprandial period. This is one of the first studies to assess cognition in patients with T2DM utilizing a multimeal paradigm. The present findings are consistent with studies showing an LGI breakfast is associated with cognitive benefits in prediabetes and T2DM relative to an HGI breakfast [7,8]. A closer look at the GCP data indicates that there was a general decline over the morning for the HGIP, which was attenuated by the LGIP, culminating in significantly better performance at 11:40 for the LGIP relative to the HGIP. This indicates that the LGIP may benefit cognition by alleviating the natural decline in cognition over the morning and by maintaining performance. This effect has been previously observed with LGI breakfasts compared with HGI breakfasts over the morning period [10,25–29] and is supported by findings from a meta-analysis that showed benefits only in the late postprandial period of 120 min or later [5]. It is important to acknowledge here that despite the correction for type 1 error, the large number of independently analyzed outcome measures increases the chances of these findings being a type 1 error.

Interestingly, the period before lunch when cognitive benefits were observed did not coincide with glycemic differences between the conditions. Indeed, this asynchrony between cognitive and glycemic effects has been observed previously in healthy young adults [8,25]. This indicates that mechanisms other than immediate blood glucose concentrations may account for some cognitive effects. For example, secondary mechanisms associated with insulin or FFA concentrations could be contributing to the cognitive effects. For example, it is possible that insulin concentrations were higher in the HGIP 2–3 h after breakfast; however, this remains speculative in the absence of direct assessment. In support, a recent study in healthy adults assessing insulin and glucose following LGI and HGI breakfasts showed greater differences in the insulin response 2 h postprandially [26], and there are well-established links between insulin sensitivity in the brain and cognition [27]. Furthermore, low carbohydrate and low glycemic load (GL) meals that produce a lower insulin response relative to high GL meals, can lead to a lower insulin-to-glucagon ratio, which has been associated with higher late postprandial circulating metabolic fuel as assessed by total FFAs, glucose, and ketones [28]. This increase in late postprandial energy availability after LGI relative to HGI could account for the observed cognitive benefits in the present study immediately before lunch, although further research is required to explore whether total circulating metabolic fuel is associated with cerebral neuronal function in the postprandial period. The absence of postprandial glycemic differences to breakfast could be due to a second meal effect from the standardized evening meal. Baked beans typically have a low GI, which may have protected against glycemic excursions following breakfast. Future studies may benefit from a high GI evening meal that is more likely to facilitate glycemic differences the following day.

Differences in mood state may also have played a role in the cognitive effects in the morning. For example, the interactions indicated that hunger and sleepiness were higher for the HGIP prior to lunch; however, the pairwise comparisons were NS. This is partially consistent with evidence that lower GI meals are associated with reduced feelings of hunger and increased satiety relative to high GI meals [14,15], and this could indeed affect cognition; however, exploring the direct impact of mood on cognitive outcomes was beyond the scope of this research. Greater sleepiness has been associated with fluctuating insulin and glucose concentrations in type 1 diabetes [29], which could explain the patterns for greater sleepiness here for the HGIP. However, much of this remains speculative and requires further investigation, particularly given that these effects were only observed prior to lunch. Regarding other mechanisms, recent data in healthy adults points toward the possibility that the glycemic response to breakfast may impact cerebral blood flow. More specifically, relative to a low GI breakfast, a high GI breakfast was shown to attenuate elements of perfusion velocity—dynamic cerebral autoregulation (dCA) measured in the middle cerebral artery using transcranial Doppler ultrasonography [26]. The dCA is a process that enables homeostasis in cerebral blood flow in the presence of rapid fluctuations in general cerebral perfusion and blood pressure. Impairments in dCA are present in T2DM [30], and inefficient dCA may negatively impact cognitive function. It is possible that a mechanism linked to dCA could have contributed to the present cognitive benefits following the LGIP in the late morning period, particularly given that there are greater fluctuations in blood pressure generally in the morning [31]. Further research assessing dCA concomitantly with cognitive function and the glycemic response in the postprandial period would be of interest.

It was evident in the present study that there were no cognitive or mood effects of the meal profiles in the afternoon, either during the postprandial response to lunch or following an afternoon snack. The simplest conclusion is that there are limited effects of glycemic response and associated physiological processes on cognition during this phase of the day. It is possible that the systematic application of type 1 error correction using the Bonferroni method to the analysis models could lead to type 2 error. It is also possible that our study was underpowered, given that the power calculation was based on a cognitive performance measure that was not assessed in this study. Variation in cognitive measures between studies in this field significantly limits the ability to use the exact same task when calculating a-priori effect size. The absence of other studies exploring the late afternoon phase following glycemic nutritional interventions means further work is required. However, null effects in the GI cognitive field are not unusual in studies with healthy adults [32,33], and some studies show that GI manipulations at lunch have no effect on cognition in the afternoon [34]. One consideration here is the cognitive post-lunch dip, which is a well-known phenomenon whereby cognition and subjective alertness decline in the immediate postprandial period following lunch, although the extent to which this is driven by nutritional intake or time of day remains under debate [35]. It is possible that any subtle effects of the meal manipulations were hidden by a larger post-lunch dip effect. Indeed, cognitive performance appears to be worse between 1.5 and 3 h post lunch relative to other times of day, as shown in FIGURE 3, FIGURE 4. Extending the test day into the evening and incorporating an evening meal in the multimeal paradigm would offer the possibility to explore cognitive effects that are outside of the post-lunch dip window. It would also be of interest to extend the paradigm to consecutive days, which would be informative from the perspective of the application of dietary change to an everyday environment. Indeed, there is evidence that long term adoption of diets designed around low GI foods, which have favorable glycemic outcomes such as improvements to HbA1C, is beneficial for mood and cognition after several months [36,37].

There are a number of limitations to this research. The absence of blood samples renders mechanistic explanations relating to insulin, FFAs, or other plasma characteristics speculative. It would be useful to compare the cognitive performance and glycemic responses of the T2DM sample to a healthy age-matched control group. This would serve 2 purposes; it would enable characterization of the degree of cognitive impairment in this T2DM sample, and it would indicate the relative effectiveness of the LGIP for producing a glycemic response akin to a healthy adult. Nutritional effects on cognitive function are most likely in those with greater capacity for improvement, such as those with more severe T2DM, or in cases where the intervention is particularly effective at benefiting a physiological response, in this case, the glycemic response. Assessing these parameters is not directly possible without a healthy control group. It is noteworthy, though, that the glycemic response to the breakfasts did not differ in the period up to lunch. This indicates that the initial breakfast meal was not effective in producing a differential response between the 2 conditions, which could explain the absence of effects later in the day, that is, a null second meal cognitive effect. Perhaps the breakfast meal is critical for determining cognitive function throughout the remainder of the day. Future studies with a multimeal paradigm would benefit from the inclusion of a breakfast that produces a clear physiological difference between the comparator conditions. Previous research also shows that nutritional interventions in T2DM are most effective when the cognitive demand is high, and the task is verbal memory based [7,8,21]. Here demand was manipulated by merging an attention task with an executive function task (the CRP and RVIP, respectively). The desired demand effect was achieved as exemplified by the data shown in Table 3 that performance was worse for the CRT-RVIP merged task (mean = 13, SD 0.6) compared with the RVIP task (mean = 18, SD = 0.6); however, there were no differences between the conditions for this task. The merged task perhaps lacked a suitable number of targets to achieve sensitivity (20 targets), and the absence of a measure of verbal memory is a notable omission. Indeed, reviews report that verbal memory is especially sensitive to impairments in glucose regulation [21,38]. The reason for this omission was practical; performance on multiple verbal memory tests over the course of the day would have been affected by significant interference and carryover effects. A limitation of a crossover design is the potential for carryover effects, particularly for cognitive function. A formal analysis of carryover effects was not undertaken due to insufficient statistical power to include the order in the models. Although counterbalancing can, to some extent, serve to minimize the impact of carryover effects in the analysis of group differences, the possibility of carryover effects remains a potential source of unexplained variance in the current study, which may be masking or magnifying group differences. Finally, variations in fiber content are almost inevitable when designing equicaloric macronutrient-matched meals to produce different glycemic responses. This creates a limitation in that any effects could be attributed to fiber or secondary processes associated with the digestion of fiber, particularly for satiety, which could explain the subtly higher concentrations of fullness for the LGIP. For example, digestive processes involving the gut microbiome can generate short-chain fatty acids, which have been implicated in the gut-brain axis as a mechanism influencing cognition function and mood [39]. Interestingly, ingestion of medium-chain triglycerides has been shown to improve cognition during hypoglycemia in patients with intensively treated type 1 diabetes [40]. It is also plausible that a diet generating a lower glycemic profile may be beneficial for the integrity of the gut barrier. Poor gut barrier integrity can lead to systemic inflammation, which can increase blood-brain barrier (BBB) permeability which is associated with neurodegeneration [41]. Indeed, it has been speculated that cognitive impairments in type 2 diabetes are associated with reduced BBB permeability [42]; therefore, if a dietary intervention can improve BBB integrity (for example, an LGIP), then this could lead to cognitive benefits.

In summary, this study shows that a multimeal paradigm using low GI foods over breakfast, lunch, and an afternoon snack broadly producing a lower glycemic response is associated with modest benefits for cognitive function in patients with noninsulin-dependent T2DM, relative to a meal profile with HGI foods producing a higher glycemic response. For most cognitive outcomes and measures of mood and satiety, there were no differences between the conditions. Interestingly, the benefits for global cognitive and executive functions were only observed ∼2.5–3 h after breakfast, immediately prior to lunch, at a point when the glycemic response was not different between the 2 conditions. Further research is required to explore possible mechanisms and explore the utility of a longer multimeal paradigm for cognitive and mood benefits in patients with T2DM and other metabolic disorders.

## Author contribution

The authors’ responsibilities were as follows–MG, DL, and JL: designed the research; MG and DL: wrote the manuscript; JL: edited the manuscript; MG: collected and analyzed the data; and all authors: read and approved the final manuscript.

## Conflict of intrest

The authors report no conflicts of interest.

## Data Availability

Data described in the manuscript will be made available upon request to the corresponding author

## Funding

The authors reported no funding was received for this study.
